# Assessment of inter- and intra-operator cephalometric tracings on cone beam CT radiographs: comparison of the precision of the cone beam CT versus the latero-lateral radiograph tracing

**DOI:** 10.1186/2196-1042-15-1

**Published:** 2014-01-06

**Authors:** Giampietro Farronato, Sara Salvadori, Francesca Nolet, Alessandro Zoia, Davide Farronato

**Affiliations:** Orthodontics Department, University of Milan, Milan, Italy

**Keywords:** Cephalometric assessment, Cone beam computer tomography, Latero-lateral teleradiography

## Abstract

**Background:**

In this study we aimed at quantifying the possible errors which may occur when assessing specific reference planes and linear derivants on cephalometric radiographs traced manually and digitally. Furthermore, we have compared the precision of the tracings according to both the two- and three-dimensional (2D and 3D respectively) techniques and between clinicians.

**Findings:**

We have obtained via cone beam computed tomography (CBCT) archive of the orthodontics department of the University of Milan 20 cone beam CT radiographs from which we have obtained 20 latero-lateral radiographs. Five independent clinicians referred to as A, B, C, D, E have been randomly selected to trace both radiographs maintaining the same working and lighting conditions to minimize the possibility of operator- and environment-dependent errors from occurring. The results have been statistically assessed by Student’s *t* test. The comparison of the data gathered from the tracings in 2D and 3D shows that certain measurements have statistically significant differences. Particularly, the difference in the measurements of the sagittal dimension of the mandible and the anterior and posterior nasal spines has resulted to be statistically significant. The results of the intra-operator comparison proved that the 3D technique is extremely precise.

**Conclusion:**

Our study determines that the 3D technique allows to obtain more precise results and with several advantages when compared to the conventional technique such as a true representation of the anatomical structures, less risk of errors occurring due to clinician skills and absence of overlapping anatomical structures.

## Findings

### Introduction

Conventional cephalometry has been used since the early 1930s as the standard procedure to assess discrepancies in the dento-alveolar and skeletal relationships. It is widely used to assess the changes that occur post-treatment and to evaluate growth [1–5]. The three X-ray projections onto which the traditional cephalometric analysis is based are the postero-anterior teleradiography, the axial projection and the latero-lateral teleradiography. However, the conventional cephalometric approach encounters several limitations such as it is a two-dimensional (2D) representation of the three-dimensional (3D) structures [6–9]. Therefore, the reliability of the cephalometric analyses depends on the correct projection and identification of errors [6–9].

To avoid such problems, cone beam computed tomography (CBCT) has been successfully introduced and used in dentistry as it represents the true 3D morphology of the skeletal structures of the cranium [10–16]. In addition, CBCT has a reduced radiation exposure when compared to multislice CT and it can also be used to assess orthodontic patients [17, 18].

In this study we assessed the number of possible errors that may occur when tracing cephalometric radiographs using the three-dimensional technique of the University of Milan compared with the precision of the radiographic position of linear measurements obtained via the tracings of the two-dimensional radiographs. We also assessed the reliability of the clinicians as we compared their own tracings after 2 months.

To carry out such assessment, we compared the precision in which different clinicians and the same clinician, but at different times, obtained specific cephalometric measurements according to the method used (either the two-dimensional or three-dimensional technique).

## Materials and methods

Twenty cone beam CT radiographs have been selected and from these, 20 corresponding latero-lateral teleradiographs have been obtained (two-dimensional technique) with the use of the software Mimics^®^ Materialise (Materialise HQ, Leuven, Belgium). The cases have been randomly selected from the archives of the orthodontics department of the University of Milan. The patients were 12 females and eight males and their ages ranged from 8 to 16 years (mean 12.9 ± 1.7 years). The patients have been informed of the research and authorization has been obtained from both patients and parents.

To decrease the risk of possible clinician-dependent errors occurring during the tracing of the cephalometric radiographs, we have maintained the same specific working conditions for each clinician. For instance, the clinicians had the same training and were all graduates from the University of Milan and hence had similar levels of understanding of the principles of cephalometric analyses.

All patients were undergoing treatment at the orthodontic department of the Dentistry University of Milan and all had had a cone beam CT radiograph using the I-Cat Classic^®^ system (Imaging Science International, Hatfield, PA, USA).

The manual tracing of the traditional latero-lateral teleradiographs was performed on fine grain 0.003 in. transparent acetate papers (orthotrace; Rocky Mountains Orthodontics, Denver, CO, USA) using a 0.3-mm lead pencil. The Unitek cephalometric protractor (Unitek, Allerum, Sweden) has been used that has a standard resolution of 1 mm and 1°.

The tracing process was conducted in a darkened room using a screen viewing box.

The CBCT radiographs of the selected patients have been assessed according to the three-dimensional cephalometric analyses of the University of Milan using the software Mimics^®^ Materialise.

Six specific reference planes have been selected and assessed which were the following:

- S-N: orientation of the anterior cranial base
- SNP-A: sagittal dimension of the upper maxillary process
- GO-ME: sagittal dimension of the mandible
- N-SNA: anterior superior vertical dimension
- SNA-ME: anterior inferior vertical dimension
- N-ME: total anterior vertical dimension

The study was carried out in July to August 2013 at (time 1 = T1) by five selected clinicians which we will refer to as A, B, C, D and E. The clinicians were randomly selected and were all orthodontists working in the orthodontics department of the University of Milan who had graduated from the University of Milan. Each has manually traced 20 cephalometric radiographs and measured six reference planes according to the two-dimensional technique. The same clinicians then traced, according to the three-dimensional cephalometric technique, the corresponding 20 cone beam CT radiographs.

After 2 months (time 2 = T2), the five clinicians (A, B, C, D, E) have repeated the same tracings using both the two-dimensional and three-dimensional techniques and they re-recorded, for each radiograph, the same six reference planes.

The statistical analyses of the data have been measured using the Student’s *t* test and statistical significance was set at *p* < 0.05. Confidence intervals were determined at 95%.

The data has been inserted into the statistical programme SPSS^®^ statistical package 17.00 for Windows (IBM Corporation, Sommers, NY, USA).

## Results

**Figure 1:**
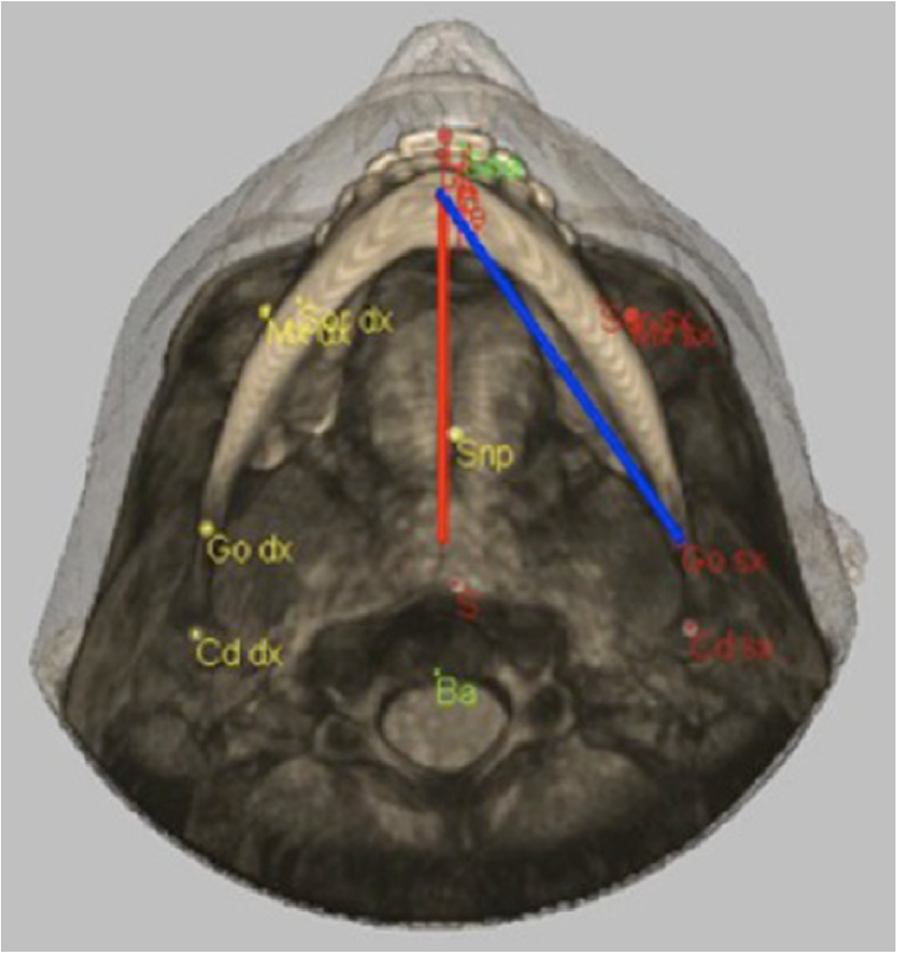
**Reference planes: the anterior and posterior nasal spines and the GO-ME plane.** The red line represents the GO-ME plane according to the 2D technique. The blue line represents the GO-ME plane according to the 3D technique.

Below is the comparison of the measurements obtained by the different clinicians at T1 and T2 according to both 2D and 3D techniques (Table 1) and the comparison of the tracings according to the 3D and 2D techniques by the same clinician at different times. The reference planes, which appeared to vary more frequently, were the anterior and posterior nasal spines and the GO-ME plane (Figure 1).

**Table 1:** **Summary of the results of the comparison of 3D with 2D techniques**

Patient	Reference planes					
	S-N	SNP-A	GO-ME	N-SNA	SNA-ME	N-ME
GA			X		X	X
BM		X	X	X		
CF		X	X			
GS	X		X			
VM		X	X			X
NG	X	X	X			
PA	X	X	X		X	
RA		X	X			
RM			X	X	X	X
SA	X	X	X	X	X	
GS		X	X		X	
MV			X			X
SC		X	X	X	X	
SM		X	X	X		
TE		X	X			
RE	X	X	X	X	X	
GC		X	X			
RN		X	X			
PA	X	X	X			X
GA	X		X	X		

Comparison of the reference planes which have been found to be statistically significant when comparing the 3D technique with 2D technique in 20 patients.

### Patient GA

The 3D versus 2D comparison assessed by Student’s *t* statistical test where *p* < 0.05 proves to be significant for the reference planes GO-ME, SNA-ME and N-ME. The 3D versus 3D comparison assessed by Student’s *t* test where *p* < 0.05 was not proved to be statistically significant. The 2D versus 2D comparison was not proved to be statistically significant.

### Patient BM

The 3D versus 2D comparison assessed by Student’s *t* test where *p* < 0.05 was proved to be statistically significant for the reference planes GO-ME, SNP-A and N-SNA. The 3D versus 3D comparison assessed by Student’s *t* test where *p* < 0.05 was not proved to be statistically significant. The 2D versus 2D comparison was not proved to be statistically significant.

### Patient CF

The 3D versus 2D comparison assessed by Student’s *t* test where *p* < 0.05 was proved to be statistically significant for the reference planes GO-ME and SNP-A.

The 3D versus 3D comparison assessed by Student’s *t* test where *p* < 0.05 was not proved to be statistically significant. The 2D versus 2D comparison was not proved to be statistically significant.

### Patient GS

The 3D versus 2D comparison assessed by Student’s *t* test where *p* < 0.05 was proved to be statistically significant for the reference planes GO-ME and S-N.

The 3D versus 3D comparison assessed by Student’s *t* test where *p* < 0.05 was not proved to be statistically significant. The 2D versus 2D comparison was not proved to be statistically significant.

### Patient MV

The 3D versus 2D comparison assessed by Student’s *t* test where *p* < 0.05 was proved to be statistically significant for the reference planes GO-ME, SNP-A and N-ME.

The 3D versus 3D comparison assessed by Student’s *t* test where *p* < 0.05 was not proved to be statistically significant. The 2D versus 2D comparison was not proved to be statistically significant.

### Patient NG

The 3D versus 2D comparison assessed by Student’s *t* test where *p* < 0.05 was proved to be statistically significant for the reference planes GO-ME, S-N and SNP-A.

The 3D versus 3D comparison assessed by Student’s *t* test where *p* < 0.05 was not proved to be statistically significant. The 2D versus 2D comparison was not proved to be statistically significant.

### Patient PA

The 3D versus 2D comparison assessed by Student’s *t* test where *p* < 0.05 was proved to be statistically significant for the reference planes GO-ME, S-N, SNA-ME and SNP-A.

The 3D versus 3D comparison assessed by Student’s *t* test where *p* < 0.05 was not proved to be statistically significant. The 2D versus 2D comparison was not proved to be statistically significant.

### Patient RA

The 3D versus 2D comparison assessed by Student’s *t* test where *p* < 0.05 was proved to be statistically significant for the reference planes GO-ME and SNP-A.

The 3D versus 3D comparison assessed by Student’s *t* test where *p* < 0.05 was not proved to be statistically significant. The 2D versus 2D comparison was not proved to be statistically significant.

### Patient RM

The 3D versus 2D comparison assessed by Student’s *t* test where *p* < 0.05 was proved to be statistically significant for the reference planes GO-ME, SNA-ME, N-ME and N-SNA.

The 3D versus 3D comparison assessed by Student’s *t* test where *p* < 0.05 was not proved to be statistically significant. The 2D versus 2D comparison was not proved to be statistically significant.

### Patient SA

The 3D versus 2D comparison assessed by Student’s *t* test where *p* < 0.05 was proved to be statistically significant for the reference planes GO-ME, S-N, SNA-ME, SNP-A and N-SNA.

The 3D versus 3D comparison assessed by Student’s *t* test where *p* < 0.05 was not proved to be statistically significant. The 2D versus 2D comparison was not proved to be statistically significant.

### Patient GS

The 3D versus 2D comparison assessed by Student’s *t* test where *p* < 0.05 was proved to be statistically significant for the reference planes GO-ME, SNA-ME and SNP-A.

The 3D versus 3D comparison assessed by Student’s *t* test where *p* < 0.05 was not proved to be statistically significant. The 2D versus 2D comparison was proved to be statistically significant for the reference values S-N and N-ME.

### Patient MV

The 3D versus 2D comparison assessed by Student’s *t* test where *p* < 0.05 was proved to be statistically significant for the reference planes GO-ME and N-ME.

The 3D versus 3D comparison assessed by Student’s *t* test where *p* < 0.05 was not proved to be statistically significant. The 2D versus 2D comparison was not proved to be statistically significant.

### Patient SC

The 3D versus 2D comparison assessed by Student’s *t* test where *p* < 0.05 was proved to be statistically significant for the reference planes GO-ME, SNA-ME, SNP-A and N-SNA.

The 3D versus 3D comparison assessed by Student’s *t* test where *p* < 0.05 was not proved to be statistically significant. The 2D versus 2D comparison was proved to be statistically significant for the reference value N-ME.

### Patient SM

The 3D versus 2D comparison assessed by Student’s *t* test where *p* < 0.05 was proved to be statistically significant for the reference planes GO-ME, SNP-A and N-SNA.

The 3D versus 3D comparison assessed by Student’s *t* test where *p* < 0.05 was not proved to be statistically significant. The 2D versus 2D comparison was proved to be statistically significant for the reference values N-ME.

### Patient TE

The 3D versus 2D comparison assessed by Student’s *t* test where *p* < 0.05 was proved to be statistically significant for the reference planes GO-ME and SNP-A.

The 3D versus 3D comparison assessed by Student’s *t* test where *p* < 0.05 was not proved to be statistically significant. The 2D versus 2D comparison was proved to be statistically significant for the reference values SNP-A and N-SNA.

### Patient RE

The 3D versus 2D comparison assessed by Student’s *t* test where *p* < 0.05 was proved to be statistically significant for the reference planes GO-ME, SNA-ME, SNP-A and N-SNA.

The 3D versus 3D comparison assessed by Student’s *t* test where *p* < 0.05 was not proved to be statistically significant. The 2D versus 2D comparison was proved to be statistically significant for the reference values S-N and SNP-A.

### Patient GC

The 3D versus 2D comparison assessed by Student’s *t* test where *p* < 0.05 was proved to be statistically significant for the reference planes GO-ME and SNP-A.

The 3D versus 3D comparison assessed by Student’s *t* test where *p* < 0.05 was proved to be statistically significant for the value S-N. The 2D versus 2D comparison was proved to be statistically significant for the values N-SNA.

### Patient RN

The 3D versus 2D comparison assessed by Student’s *t* test where *p* < 0.05 was proved to be statistically significant for the reference planes GO-ME and SNP-A.

The 3D versus 3D comparison assessed by Student’s *t* test where *p* < 0.05 was not proved to be statistically significant. The 2D versus 2D comparison was proved to be statistically significant for the reference values GO-ME, N-ME and N-SNA.

### Patient PA

The 3D versus 2D comparison assessed by Student’s *t* test where *p* < 0.05 was proved to be statistically significant for the reference planes GO-ME, S-N, SNP-A and N-ME.

The 3D versus 3D comparison assessed by Student’s *t* test where *p* < 0.05 was proved to be statistically significant for the value N-SNA. The 2D versus 2D comparison was proved to be statistically significant for the reference values N-SNA.

### Patient GA

The 3D versus 2D comparison assessed by Student’s *t* test where *p* < 0.05 was proved to be statistically significant for the reference planes GO-ME, S-N and N-SNA-ME.

The 3D versus 3D comparison assessed by Student’s *t* test where *p* < 0.05 was not proved to be statistically significant. The 2D versus 2D comparison was proved to be statistically significant for the reference values SNP-A and N-SNA.

## Discussion

Our findings suggest that generally the five clinicians gave a subjective interpretation of the position of the reference planes on the tracings regardless of the technique used, even though they have had the same training and had the same skills.

However, none of them obtained measurements, which were above or below the average, but they have, at times, overestimated or underestimated certain measurements when using both techniques. The comparison of the data gathered from the tracings of the 2D and 3D radiographs shows that several measurements have statistically significant differences.

**Figure 2:**
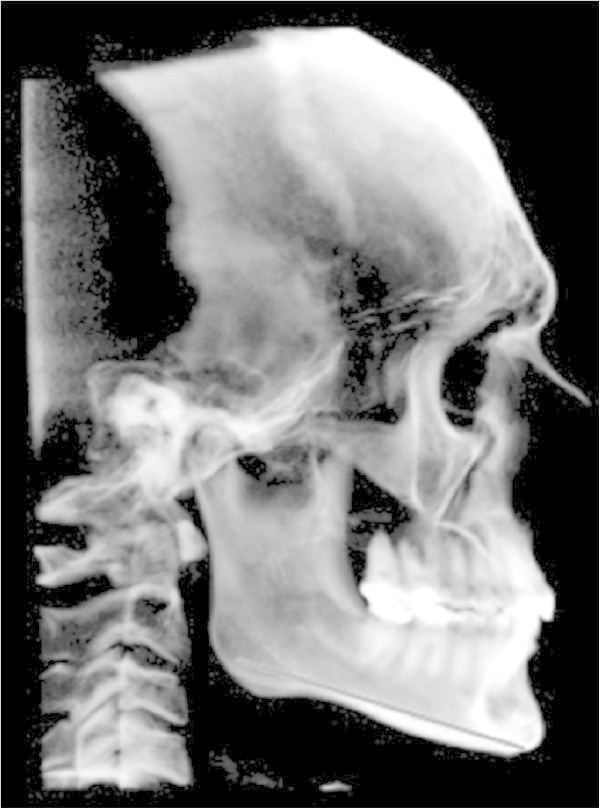
**Latero-lateral view of the GO-ME measurement.**

Particularly, the measurement of the reference plane GO-ME results to be always statistically significant (Figure 2). This difference can be justified by the fact that the measurements obtained from the 2D technique determine a projection of the points, which in reality are placed on different planes. Differently, the planes measured using the 3D technique are based on a real measurement (an oblique plane on the sagittal axis) rather than prospective ones [6–8]. Hence, the real measurement for the two-dimensional technique is represented by its projection with the consequent result that measurements obtained from the 2D radiographs are less accurate than the ones obtained from the 3D radiographs [19, 20].

**Figure 3:**
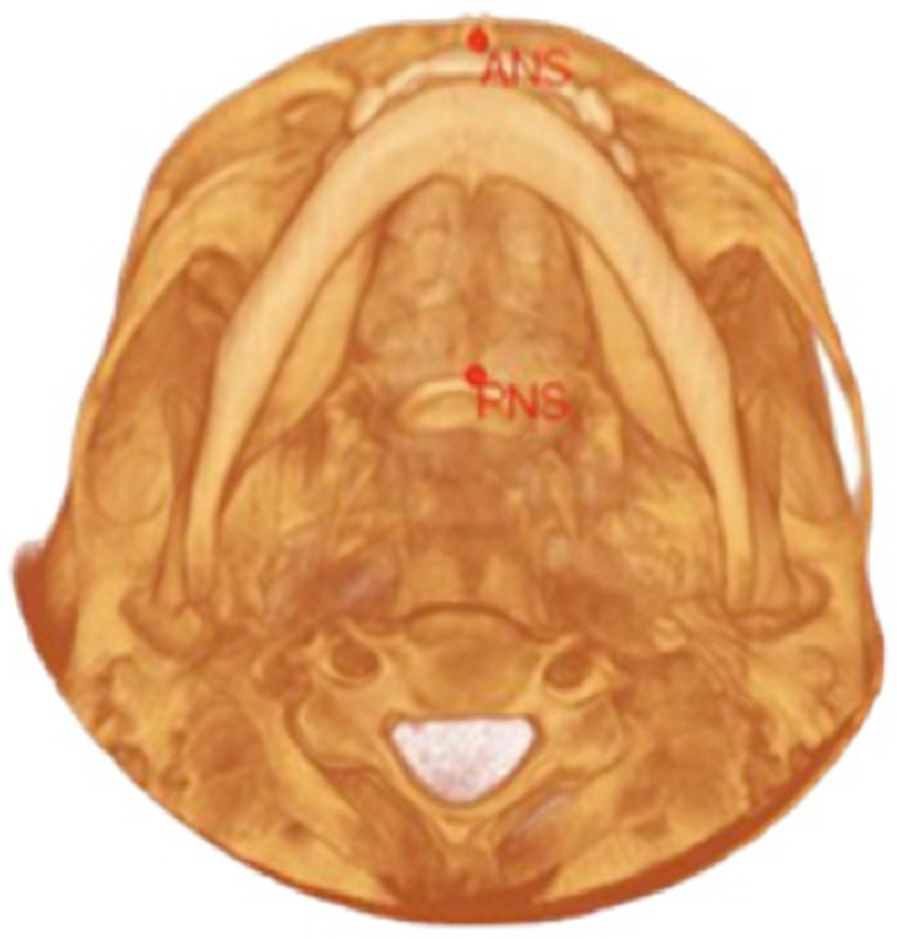
**Axial projection of the cranium.** Location of the anterior (ANS) and posterior nasal spine (PNS) obtained from a CBCT radiograph.

The other values in which we noticed a variation, which was statistically significant between the two techniques, were all values in which it is necessary to identify the anterior nasal spine (ANS) or the posterior nasal spine (PNS) (Figure 3). These points are, in fact, particularly difficult to identify with the 2D technique due to the numerous overlapping anatomical structures and complication, which do not occur when using the 3D technique [20].

Regarding the comparison of the linear measurements obtained by the same clinician on the tracings after 2 months, we have noticed that each clinician has traced the 2D cephalometric radiographs obtaining important variations and differences between the first time (T1) and after 2 months (T2). Each clinician has obtained, for the majority of the radiographs, the precise position of the linear measurements both times (initially and after 2 months) when tracing the 3D radiographs (Tables 2 and 3).

**Table 2:** **Summary of the intra-operator results for the 3D technique (3D versus 3D)**

Patient	Reference planes					
	S-N	SNP-A	GO-ME	N-SNA	SNA-ME	N-ME
GA						
BM						
CF						
GS						
VM						
NG						
PA						
RA						
RM						
SA						
GS						
MV						
SC						
SM						
TE						
RE						
GC	X					
RN						
PA				X		
GA						

Comparison of the reference planes, which have been found to be statistically significant when comparing the precision of the tracings in the 3D technique by the same clinicians at T1 and T2.

**Table 3:** **Summary of the intra-operator results according to the 2D technique (2D versus 2D)**

Patient	Reference planes					
	S-N	SNP-A	GO-ME	N-SNA	SNA-ME	N-ME
GA						
BM						
CF						
GS						
VM						
NG						
PA						
RA						
RM						
SA						
GS	X					X
MV						
SC						X
SM						X
TE		X		X		
RE	X	X				
GC				X		
RN			X	X		X
PA				X		
GA		X		X		

Comparison of the reference planes which have been found to be statistically significant when comparing the precision of the tracings in the 2D technique by the same clinicians at T1 and T2.

In conclusion, we have determined that the use of the computer for the tracings of the 3D radiographs obtains less measurement errors and overall much more precise cephalometric analyses. The 3D technique has thus several advantages when compared to the conventional technique such as a true representation of reality, a lesser risk of operator-dependent errors from occurring and absence of overlapping anatomical structures [21]. However, to be able to confirm such results, further research is needed on a bigger sample.
